# Has decentralisation affected child immunisation status in Indonesia?

**DOI:** 10.3402/gha.v7.24913

**Published:** 2014-08-25

**Authors:** Asri Maharani, Gindo Tampubolon

**Affiliations:** 1 Medical Faculty, University of Brawijaya, Indonesia; 2 Institute for Social Change, University of Manchester, United Kingdom

**Keywords:** Fiscal decentralisation, immunisation status, Indonesia, multilevel model, multiple imputation

## Abstract

**Background:**

The past two decades have seen many countries, including a number in Southeast Asia, decentralising their health system with the expectation that this reform will improve their citizens’ health. However, the consequences of this reform remain largely unknown.

**Objective:**

This study analyses the effects of fiscal decentralisation on child immunisation status in Indonesia.

**Design:**

We used multilevel logistic regression analysis to estimate these effects, and multilevel multiple imputation to manage missing data. The 2011 publication of Indonesia's national socio-economic survey (Susenas) is the source of household data, while the Podes village census survey from the same year provides village-level data. We supplement these with local government fiscal data from the Ministry of Finance.

**Results:**

The findings show that decentralising the fiscal allocation of responsibilities to local governments has a lack of association with child immunisation status and the results are robust. The results also suggest that increasing the number of village health centres (*posyandu*) per 1,000 population improves probability of children to receive full immunisation significantly, while increasing that of hospitals and health centres (*puskesmas*) has no significant effect.

**Conclusion:**

These findings suggest that merely decentralising the health system does not guarantee improvement in a country's immunisation coverage. Any successful decentralisation demands good capacity and capability of local governments.

In the past 20 years, decentralisation has been considered by many policy-makers to be an important strategy to improve the performance of health systems, including those in South East Asian countries such as Thailand (1, 2), Malaysia (3), The Philippines (4), Vietnam (4), and Indonesia (5). The expectation of these policy-makers is that in the health sector, decentralisation will improve efficiency, service delivery innovation, quality, and equity in healthcare, which in turn will improve the health status of the population (6, 7). Under decentralisation, central government devolves responsibility for health service delivery to local governments, including the authority to carry out local planning, procurement of equipment, financing, and evaluation (8–11).

Based on the type of authority devolved, decentralisation is made up of: political, administrative, and fiscal (12, 13). Political decentralisation refers to the degree to which central government allows local governments to undertake the political functions of governance. Administrative decentralisation transfers the administration and delivery of public services from central to local governments, while fiscal decentralisation is designed to increase local government control of revenue. As fiscal decentralisation should result in expenditure being matched more closely to local needs and preferences, the expectation is that local government will increase the efficiency of public service provision while at the same time increasing the promotion of accountability (14). Furthermore, fiscal decentralisation allows local governments to raise revenue, for example, through their ability to tax or to receive grants (15). The focus in this paper is on fiscal decentralisation as this is the most important step in the overall decentralisation process (16).

Previous studies, both multi-country and single-country, have identified a positive association between fiscal decentralisation and health outcomes. For example, a study which used panel data from a number of low- and high-income countries found that fiscal decentralisation was inversely related to infant mortality rate and concluded that the marginal benefit obtained by fiscal decentralisation is greater for low-income countries (17). It also argued that economic development in low-income countries increases the institutional capacity of local authorities faster than that of central government. A number of single-country studies have presented similar results. For example, a study using a large panel data of Argentine provinces demonstrated a negative relationship between fiscal decentralisation and infant mortality rates (18). Another study using an index of fiscal decentralisation based on spending and revenue measures for rural villages in India concluded that decentralisation reduces infant mortality rates and that the effectiveness of fiscal decentralisation is commensurate with the degree of political decentralisation (16). It also mentioned the role which local authority capacity plays in the successful utilisation of a decentralised budget. Finally, comparable results highlighting the relationship between fiscal decentralisation and infant mortality rates have been presented in recent studies of China (19), Spain (20), and Colombia (21).

This evidence on the association between fiscal decentralisation and health outcomes tends to be based on aggregate analysis, with district and country as the units of analysis. However, it is well-known that such analysis risks the invalid transfer of aggregate results to individuals (22). This risk may result in biased inference due to loss of information when using ecological correlations as a replacement for individual correlations. We decided therefore to use a multilevel model, and our study contributes to the existing literature by distinguishing individual as well as local government determinants in our analysis. By accounting for this multilevel structure of individuals within districts, we were able to investigate whether the effect of local government conditions on individual health outcomes varies between local governments. This meant that the effect of fiscal decentralisation on individual health outcomes could thus be tested appropriately. Moreover, by combining contextual and individual determinants, we are able to examine the effect of local government fiscal capacity on the promotion of health status in Indonesia.

The aim of this study was to examine the consequences of fiscal decentralisation specifically on child immunisation status in Indonesia. The reason for this focus was firstly because immunisation is accepted as a proxy for similar public services, such as family planning and other preventive services. Furthermore, immunisation is the most cost-effective health intervention in terms of reducing both the morbidity of vaccine-preventable diseases and the child mortality rate (23, 24), increasing the significance of the general effects of decentralisation and health reform (25–27).

Evidence of the effect of decentralisation on immunisation status across countries presents various outcomes. In India, for example, a study in Kerala showed decentralisation resulted in improved access to immunisation programmes and increased Diphtheria, Pertussis, Tetanus (DPT) immunisation coverage (28). The reasons were found to be improved infrastructure (including facilities and equipment) in Kerala's healthcare institutions, and better accountability in the public healthcare system. In contrast, studies of Papua New Guinea have revealed a decrease in Bacille Calmette-Guérin (BCG) immunisation coverage among children under 1 year following decentralisation. Similarly, an immediate evaluation of decentralisation in Tanzania's Expanded Programme on Immunization (EPI) found services at district level to be of poor quality. Reasons for this included inadequate cooperation between central and local policy-makers, demoralised health service providers, a reduced number of supervision visits by EPI staff, and the improper maintenance of vaccine temperature (29). Interestingly, a cross-country study found that decentralisation has different effects in low- and middle-income countries. Decentralised low-income countries were found to have higher measles and DPT3 immunisation coverage than centralised ones, while in contrast decentralised middle-income countries have lower immunisation coverage for the same period (7).

The potentially negative effect of decentralisation on Indonesia's immunisation status has been discussed by international organisations working in health and immunisation, such as USAID and GAVI (30, 31). They have commented on the stagnation of immunisation coverage in Indonesia during the previous decade, suggesting that decentralisation has contributed to it. Before decentralisation, the government of Indonesia paid considerable attention to improve coverage of basic childhood immunisation against polio, measles, diphtheria, tetanus, pertussis, and tuberculosis. In 1977, it officially initiated EPI activities which provided basic, free immunisation for all children. Unlike most of Indonesia's maternal and child health services, this national immunisation programme was not fully decentralised. Instead the responsibility for the supply and cold chain maintenance of vaccines was retained by central government, while that for the provision of the health facilities, health professionals, and equipment needed to carry out vaccination was devolved to district governments (32). This division of responsibility has led in some cases to uncertain programme ownership (possibly exacerbated by differing priorities at local level) and has almost certainly played a part in the stagnation of immunisation coverage since decentralisation (30).

A study examining 10 districts of Java found that there has been no improvement in DPT3 immunisation coverage since decentralisation, despite the significant increase in public health expenditure. One reason for this failure is the limited analytical and planning capacity of local government representatives, who were not provided with the education and training needed to plan and implement their new areas of responsibility. The failure of decentralisation to improve child immunisation in Indonesia is not in dispute; the need to address this is urgent, and the first step is to examine the consequences of decentralisation on immunisation status.

Indonesia constitutes a particularly interesting case, not only because of the size of the country but also because of its remarkable progress in creating a decentralised system of government in a relatively short period of time. Starting in 2001, Indonesia devolved responsibilities from central to district government in almost all government administrative sectors (including health) with the aim of improving efficiency, quality, and equity of public service provision (4). Evaluating the consequences of decentralisation in Indonesia also provides lessons for other Southeast Asian countries, especially those with similar reform and reform backgrounds. Like other Southeast Asian countries, decentralisation of the health sector in Indonesia was launched in the late 1990s (before general decentralisation in 2001) following the 1997 East Asian financial crisis (4), and has wider implications throughout Southeast Asia, whose countries face a similar epidemiological challenge of tropical infectious diseases among children.

Two studies in particular have found that health sector decentralisation in Indonesia has failed to achieve its aim, and they highlight several plausible explanations for this failure (33, 34). The first explanation is that local governments only have real discretion for less than 30% of their health expenditure (33), a figure which is low compared to the average of local expenditure autonomy (58%) experienced in other developing countries (14). Another explanation is the limited capacity of local governments, which are given responsibility for funds after decentralisation but not the skills needed to utilise them appropriately (34, 35). Unlike Thailand (possibly the most successful example of decentralisation in Southeast Asia) (1), local authorities in Indonesia are not required to demonstrate sufficient capacity and commitment before receiving greater autonomy under decentralisation.

There are a number of studies which evaluate the consequences of decentralisation in Indonesia. However, their usefulness is limited by the fact that they only cover some of Java's districts. There are nearly 500 districts across Indonesia, and those outside Java tend to be poorer. Omitting districts on remote islands means these studies capture only a partial picture of the country. This adds urgency to the need to evaluate the effects of decentralisation in all districts in Indonesia, and specifically its association with child immunisation. Our study has used data sourced from multiple surveys (contextual, household, and individual) in 497 districts.

## Data and methods

### Data

This study combines data from various sources. The Indonesian national socio-economic survey (*Survei sosial ekonomi nasional*, or Susenas) in 2011 was the main source of household-level data. It provided information on a child's immunisation status as well as the characteristics of the mother and the socio-economic status of the household. Alongside Susenas, we assembled data from the 2011 national village census (*Potensi desa*, or Podes) and government fiscal information. Podes provided information on population and the number of health facilities in all villages within a district, the aggregate of which is calculated for each district. We included health facilities which provide immunisation for children: hospitals, public health centres (*Puskesmas*), and integrated health services posts (*Posyandu*).^1^ The government fiscal data was obtained from the Ministry of Finance. We linked the Susenas data to the other data sources using district codes. Taken together this data captures the nested structure of households by district.

### Immunisation status measure

The key outcome variable is complete immunisation status among children aged 12–23 months. We extracted the data on immunisation status from Susenas, in which parents are asked whether their children received each of the basic immunisations or not and the number of doses received for each. Although every immunised child receives an immunisation card recording the date of immunisation and how many they have received, the parent was not obliged to show this card to the Susenas researcher. The data were created based on the answers of the parents. We define complete immunisation status based on a child receiving each of the immunisations in the national EPI schedule (36).^2^ Children above 2 years old are not included in this study to avoid confusion with the immunisation booster schedule.

**Table 1:** Schedule of Indonesia routine immunisation

Age of administration	Antigens		
0 month	BCG	HB0	OPV0
2 months	DPT1	HB1	OPV1
3 months	DPT2	HB2	OPV2
4 months	DPT3	HB3	OPV3
9 months	Measles		

We measured child immunisation data as a binary variable (1=received complete basic immunisation; 0=not received complete basic immunisation) – complete basic immunisation is important to protect children from vaccine-preventable diseases. Incomplete immunisation (e.g. a child receiving only two shots of DPT immunisation from a series of three) means that immunity is not completely formed. The Indonesia government emphasises the importance of complete basic immunisation to eradicate these diseases and to reduce child mortality rate (37).

### Fiscal decentralisation measure

We measured fiscal decentralisation using the ratio of local public expenditure on health to total local public expenditure, and found that this measurement reflects responsible governance at the local level. The most common measure of fiscal decentralisation is the local share of total government expenditure (16, 17, 19). However, this measure conveys only a limited reflection of fiscal decentralisation, as it fails to consider the control which local authorities have over funds raised locally or other local potential resources (21, 38). A study in Colombia extended these measures by using the ratio of locally controlled health expenditure to total health expenditure. However, this measure is less suitable for the case of decentralisation in Indonesia, where local governments received funds from central government in a bulk called the balancing fund (*dana perimbangan*). It includes a general grant (*dana alokasi umum*), shared taxes, natural resource revenue shares, and a special allocation grant channel (*dana alokasi khusus*) (39). Although the transfers from central government to local government remain the dominant means of financing, earmarking is gone, and local governments have the authority to allocate the funds for each public service sector, including health. We therefore decided to use a fiscal decentralisation measure which represents the resources used to finance the health sector over all resources for which local governments have authority and also discretion on how to use these resources. We consider this measure more useful, as it captures the willingness of local governments to allocate their funds for the health sector.

### Household-level determinants

Determinants at household level consist of birth attendants, mothers’ employment status, mothers’ age, mothers’ education, and household socio-economic status. We created a dummy variable for birth attendants (1 for a child whose birth was attended by health professional – physicians, midwives and nurses – and 0 for a child whose birth was not). Employment of mothers is measured using a dummy variable (1 for employed and 0 for unemployed). We classify mothers’ age into three levels: ≤20 years, 21–30 years and>30 years, and measure their education according to the highest level of education attained, differentiated into three levels: primary, secondary, and tertiary education. Household socio-economic status is measured using household expenditure over 1 year. Household expenditure variable is entered as a log-transformed continuous variable to make the distribution more symmetric and to reduce the effect of outliers.

### District-level determinants

We used a number of determinants which measure variation in local health provision to examine contextual effects. First, we took the number of health facilities per 1,000 population to measure the availability of healthcare providers, especially in regard to immunisation services (hospitals, health centres, and village health posts). We also used the proportion of urban population, population density, and gross domestic product (GDP) per capita as district-level determinants. Similar with household expenditure variable, we entered GDP per capita as a log-transformed continuous variable.

## Methods

Our study used a multilevel logistic regression model (which we believe to be most appropriate because it considers the nested structure of households within districts), and estimated the association of fiscal decentralisation with child immunisation status in Indonesia, treating the dependent variable as binary (complete immunisation or not). The first level comprised household characteristics and district characteristics made up the second level. Considering households *i* nested in districts *j*, the model is:

$$E_{ij}*=\gamma_{00}+\Sigma \gamma_{0j}W_{j}+\Sigma \beta_{ki}X_{ij}+u_{0j}+\varepsilon_{ij}$$

with:

*E*_ij*_=logit(*P*(*E*_*ij*_=1)),

*W*_*j*_ is a set of district characteristics,

*X*_*ij*_ is a set of household characteristics,

*u*_0*j*_ are the random intercept varying over district γ_00_ with mean zero and variance $\sigma_{00}^{2}$,

ɛ_*ij*_ is normally distributed with mean zero and variance $\sigma_{\varepsilon }^{2}$.

### Missing data

Where there were missing data, we obtained multilevel multiple imputed values, which avoided the potential bias which can arise when incomplete data is mishandled (when cases are deleted, or when indicator variables are used for missing data). This also made full use of the observed data, since missing data appeared at both household and district level. Multilevel multiple imputation under missing-at-random assumption was used to estimate missing data for complete immunisation status and covariates (40). We used all predictors taken together to impute the missing values and analyse the imputed data.

## Results

**Fig. 1:**
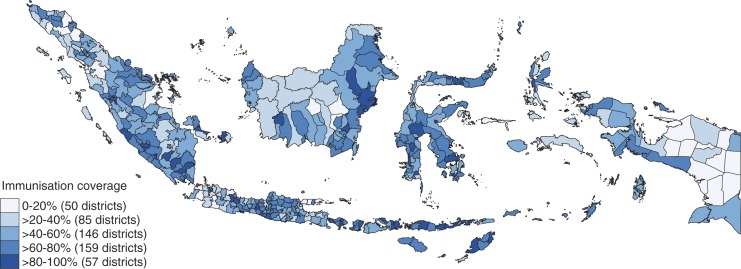
Spatial distribution of immunisation coverage among districts in Indonesia.

**Fig. 2:**
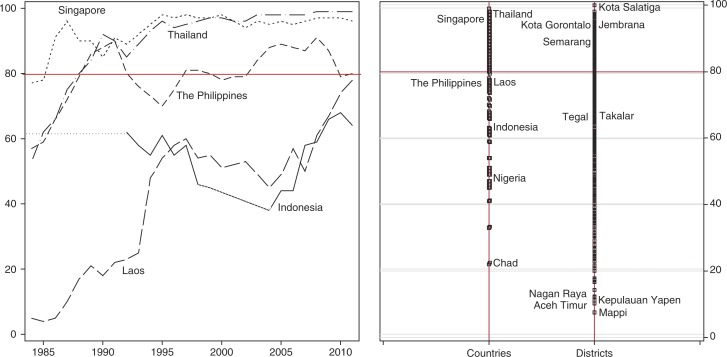
DPT3 coverage in Indonesia and selected comparators (1985–2011) and comparison with Indonesia district attainment (2011).

We begin by describing immunisation status and characteristics of both households and districts, and then present the results of the multilevel analysis of predictors of child immunisation status. The descriptive statistic (see Table 2) shows that almost half of the children in the survey did not receive complete immunisation. This means that more than a decade after decentralisation, Indonesia is missing the WHO immunisation target of 80% and thus failing to provide basic primary care services. However, this national figure masks huge variation across districts. Fig. 1 highlights this, showing that more than four out of every five children in 57 districts were covered with complete immunisation, while in 50 other districts less than one in every five children was covered. A sense of the importance of area variation in immunisation coverage can be gained from the map in Fig. 2, which highlights geographical disparities across districts and compares district attainment of DPT3 immunisation coverage in Indonesia to that of selected countries in Southeast Asia region. Overall, DPT3 coverage in Indonesia is far below that of Thailand and Singapore (often presented as examples which have over the last two decades performed well compared to other countries in the region), and performed slightly worse than the Philippines and Laos (Laos performed well below other Southeast Asia countries before the 1990s, but by 2011 it had improved significantly and performed better than Indonesia). Within Indonesia, we observed an immense variation of DPT3 coverage between districts. The three districts of Kupang, Gorontalo, and Jembrana achieved a notable public services performance, with the same DPT3 coverage as Thailand and Singapore, while at the other end of the scale, almost all the children included in the Susenas survey in Mappi, Aceh Timur, Yapen, and Nagan Raya missed complete immunisation. This wide gap of achievement between districts necessitates analysis at district and individual levels, not at national level.

**Table 2:** Descriptive statistics on household and district characteristics

	Mean (%)	SD	Missing (%)
Household characteristics			
Complete immunisation status			0.17
Child receive complete immunisation	53.36%	23.07	
Child not receive complete immunisation	46.64%		
Residential areas			0
Rural	61.55%		
Urban	38.45%		
Birth attendants			0
Non-health professional	24.31%		
Health professional	75.69%		
Mothers’ age			0
20 years	6.18%		
21–30 years	52.69%		
> 30 years	41.13%		
Mothers’ education			4.19
Primary/no education	60.56%		
Secondary	28.06%		
Higher	11.39%		
Mothers’ employment status			0.01
Unemployed	59.69%		
Employed	43.01%		
Household income (IDR 1,000)	2406.27	2456.86	0
District characteristics			
Local health expenditure as a proportion of total expenditure (%)	9.53	3.29	1.50
Hospitals/1,000 population	0.03	0.03	1.99
Health centres/1,000 population	0.23	0.20	1.99
Village health posts/1,000 population	1.39	0.60	1.99
Proportion of urban population	0.39	0.30	0
Population density	1058.98	2525.54	0
GDP per capita (IDR 1,000)	19986.8	32881.2	0
Number of children	23,766		
Number of districts	497		

**Fig. 3:**
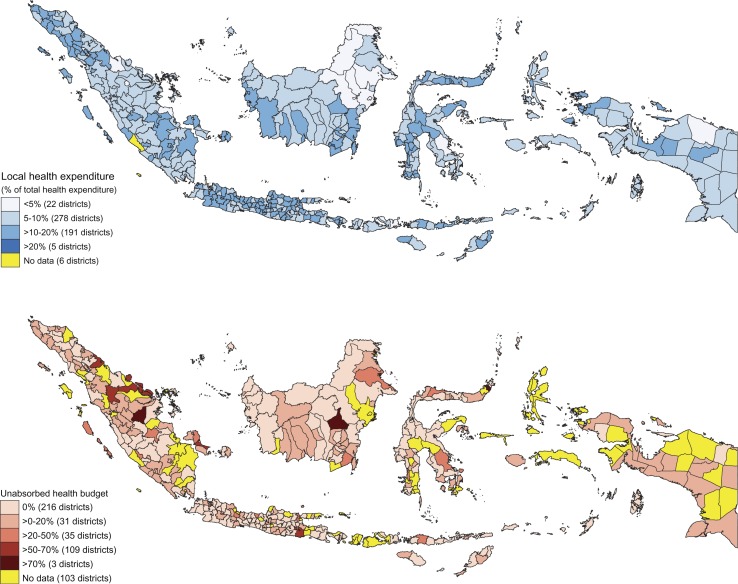
Health budget and expenditure across districts in Indonesia 2011.

A similar variation occurs in the percentage of local government expenditure allocated for healthcare, and indicates different levels of concern for the health sector (Fig. 3). Five districts prioritise health and allocate more than one-fifth of their expenditure for health, while some districts allocate less than 5%. An indication of the different capacities of local authorities to manage their health budget is shown by their utilisation of it. Less than half of all districts used all of their health budget. More than a 100 districts used less than half of their health budget and three districts leave more than 70% of their health budget unused. Details of the utilisation of this budget are shown in financial flows of local governments (Table 3). On average, most of local government revenue (86.57%) was transferred from central government. Local governments use more than 75% of the money on salaries and other operational expenditure (52.38 and 24.89%, respectively), while the expenditure for investment (facilities and infrastructure) comprises only less than 25% of total local government expenditure. This is expenditure which has the potential to contribute to an improved public health outcome, although as is clear from the literature, this is not guaranteed. Modelling the association of fiscal decentralisation with immunisation status was done next.

**Table 3:** Financial flows at district level 2010 (in percentage)

	Mean	SD	Min	Max
Revenue				
Own-source revenue	6.58	8.03	0.19	68.69
Transfer from central government	86.57	9.85	25.82	99.6
Transfer from provincial government	3.69	3.16	0	24.43
Revenue from other sources	3.15	4.25	0	21.45
Expenditure				
Salary	52.38	14.23	0	79.81
Other operational expenditure	24.89	6.87	3.89	59.12
Investment	22.39	10.81	0	58.62
Other expenditure	0.32	2.72	0	59.84

Multilevel logistic regression analysis (Table 4) was carried out using three models. The first model included only household-level determinants, while the second and the third models included both household- and district-level determinants. The main district-level determinant included in the second model is local health expenditure as a proportion of total local expenditure, while in the third model determinants are the number of hospital, health centre and village health post per 1,000 population. We used two different models to avoid double counting since local governments also spend their money on these three types of health facility. In addition of these determinants, we included proportion of urban to total population, population density, and log GDP per capita.

**Table 4:** Determinants of child's immunisation status

	Model 1	Model 2	Model 3
Household-level variables			
Residential areas			
(Rural)			
Urban	0.12 (0.04)‡	0.12 (0.04)‡	0.13 (0.04)‡
Birth attendants			
(Non-health professional)			
Health professional	0.43 (0.04)‡	0.42 (0.04)‡	0.42 (0.04)‡
Mothers’ age			
(≤20 years)			
21–30 years	0.12 (0.06)†	0.13 (0.06)†	0.12 (0.06)†
> 30 years	0.13 (0.07)†	0.13 (0.07)†	0.13 (0.07)†
Mothers’ education			
(Primary or less)			
Secondary	0.18 (0.04)‡	0.18 (0.04)‡	0.19 (0.04)‡
Higher	0.30 (0.06)‡	0.29 (0.06)‡	0.31 (0.06)‡
Mothers’ employment status			
(Unemployed)			
Employed	−0.04 (0.03)	−0.03 (0.03)	−0.04 (0.03)
Log households income	0.18 (0.03)‡	0.18 (0.03)‡	0.18 (0.03)‡
District-level variables			
Local health expenditure as a proportion of total expenditure (%)		2.03 (1.57)	
Hospitals/1,000 population			−0.65 (2.24)
Health centres/1,000 population			−0.43 (0.28)
Village health posts/1,000 population			0.54 (0.09)‡
Proportion of urban population		0.52 (0.23)†	0.74 (0.27)‡
Population density		−0.00 (0.00)‡	−0.00 (0.00)†
Log GDP per capita		−0.13 (0.07)*	−0.12 (0.07)*
Between district variance	1.09	1.05	1.01
ICC	0.25	0.24	0.23
Median odds ratio	2.71	2.65	2.61

Note: Reported are marginal effects (standard error). Sig.:

* significant at 10% or less

† significant at 5% or less

‡ significant at 1% or less.

Results from the first model showed that living in urban areas, the presence or otherwise of birth attendant, mothers’ education level, and households’ income are all statistically significant at 1%. Among these household-level determinants, it seems that the effect of having a professional birth attendant is the most influential, with children in this category having 43% higher probability to receive complete immunisation than children whose birth were not, holding all other determinants constants. Turning to other determinants, children who live in urban areas and those of better-off families are more likely to be immunised. Mothers’ characteristics also play an important role in their child immunisation status. Mothers who have only completed primary education or less are less likely to immunise their children than those with a higher level of education, while teenage mothers have a lower probability of immunising their children than older mothers. However, the effect of mothers’ employment status is small and far from statistically significant. Overall, these estimates remain consistent in each of the three models.

In the second model, the results indicate that there is insufficient evidence to reject the null hypothesis that local health expenditure as a proportion of total expenditure is not correlated with immunisation status among children. We check the plausibility of threshold effect by re-parameterising the local health expenditure proportion as tertiles and quintiles (Appendix 1). The test of joint significance indicates that both the tertiles (*χ*^2^=3.21, df=2, *p*=0.2) and the quintiles (*χ*^2^=4.93, df=4, *p*=0.29) of the local health expenditure as a proportion of total expenditure has no significant effect on immunisation status.

In the third model, the results show that increasing the number of village health post by one per 1,000 of the population improves the probability of children receiving complete immunisation by 54%. However, adding a hospital and a health centre has no significant effect. The effects of proportion of urban to total population, population density and the wealth of the district (as shown by GDP) remain consistent in the second and third models. Children living in a district with a higher proportion of urban population have a higher probability of having full immunisation. In contrast, those who live in densely populated districts have a lower probability of receiving immunisation, although the effect is minuscule in size.

As we used a multilevel logistic regression model in this study, we explain the effect between levels using median odds ratio (MOR) (41, 42). The MOR compares two children from two randomly chosen districts. In the first model, for two children with the same household-level determinants, the MOR of the child living in a district with a higher propensity of receiving immunisation to the child living in the district with a lower propensity is 2.71. This is a high odds ratio (41), suggesting that the heterogeneity is substantial. Including district-level determinants in the second and third models reduces the unexplained heterogeneity between districts to MORs of 2.65 and 2.61, respectively, which are still high. Thus, the propensity of children to receive complete immunisation varies a great deal between districts. Furthermore, the results of analysis using multilevel multiple imputed data are reasonably similar, in that they exclude all individuals with missing values (available in Appendix 2). This sensitivity analysis shows that the results are robust.

## Discussion

Indonesia launched decentralisation in 2001, devolving greater authority to local government with the aim of improving the efficiency, quality and equity of healthcare services, with the expectation that this would increase the health status of the population (4, 43). This study evaluates the consequences of fiscal decentralisation on child health by assessing childhood immunisation status across districts in Indonesia. In contrast with findings from other countries (16, 18–21), our results show that fiscal decentralisation has no statistically significant association with child immunisation outcomes. To shed more light on the failure of fiscal decentralisation in Indonesia to achieve its aim, we looked at the flow and utilisation of local government expenditure. Local governments rely on transfers from central government, which account for 87% of all their revenue (Table 3). However, the bulk of local government expenditure is spent on salaries (54%) (the central government has control over district health personnel). This means that local governments only have discretion on over 30% of central government transfer, plus any revenue they are able to raise themselves. Model 2 however showed that increasing this discretion has no bearing on child health outcomes (Table 4).

We thus turned to a different explanation, one of capability. Implementation of decentralisation does not necessarily mean that the decentralised entities can manage the system they are presented with. Several studies in Indonesia highlight the importance of local authority capability, especially in regard to planning, budgeting, and utilising their budget successfully. A study in 10 districts in West Java and East Java provinces discovered that the absence of leadership and vision among bureaucrats at local level meant they continued to implement the old system after decentralisation, rather than responding to the health problems in their area (34). Furthermore, a study of Southeast Sulawesi province reported a district allocating a mere 2% of its budget to the health sector, and that the local authorities in this sector have no planning and budgeting capability. At the same time, none of the budget was allocated for capacity building (44). Similar facts have been presented in studies of West Sumatra (45), Jambi (46), and West Kalimantan provinces (47).

Our results revealed that the ability of local governments to utilise their budget varies enormously, with more than half (57%) of the districts failing to absorb their entire health budget (Fig. 3). Even worse, three districts utilised less than 30% of their budget. Under these circumstances, it is unlikely that local government programmes will perform well. We found a wide variation in immunisation coverage, with some districts performing better and exceeding the WHO cut-off (80%), and others performing much worse. Such variation is difficult to reconcile since all district governments exercised similar discretion over expenditure after decentralisation. We concluded that this difference emphasises the importance of local government capability to manage their budget according to local needs.

There are several ways in which local government could utilise the health budget to improve health status. Increasing the number of village health posts in districts, for example, since immunisation status is found to be positively associated with the number of village health posts per 1,000 population. The district government of Jembrana is recognised as an example of one which has deployed most of its budget and has provided successful innovation in its health services following decentralisation. In 2003 it launched the *Jaminan Kesehatan Jembrana* health insurance scheme, which provides free primary healthcare services for all its citizens, on top of which, to improve the equity of access to healthcare, it provides free secondary and tertiary healthcare services for poor residents (48). Our study shows that immunisation status among the children of Jembrana district a decade after decentralisation was considerably high with 93% coverage, comparable to that of Singapore and Thailand (Fig. 2).

The importance of providing health facilities to improve healthcare is supported by household level findings. Children living in rural areas and poor households are less likely to be covered by complete immunisation, despite the government providing free immunisation services for all children. The real cost of accessing healthcare renders households with low economic status and in rural areas unable to access immunisation services, as transport and opportunity costs are not borne by the government. These costs impose a greater burden on poor households, and negatively affect their healthcare-seeking behaviour (49). This household-level finding supports the district-level findings, namely that a more even distribution of village health posts as one of immunisation providers improves immunisation coverage. Better distribution of immunisation providers decreases the distance to health providers which in turn increases immunisation status among children due to lower financial costs and shorter time needed to get to these providers. Previous studies in Nanggroe Aceh Darussalam revealed that the increasing local budget allocation for health sector has a positive impact on physical infrastructure budgets (50). However, a considerable amount (40%) of budget for the health sector was spent on public hospitals, which mainly provide curative care services (51). Our study finds that among the three types of health facilities (hospital, health centre and village health post), only village health post has significant and positive association with child immunisation status. Village health post provides promotive and preventive healthcare services and located in villages, which is more affordable than other health facilities. The budget for the health sector should be allocated more to increase the number of this type of health facility to improve immunisation coverage.

The main limitation of this study is that the analysis used cross-sectional datasets. Further study using data from several years, both before and after decentralisation, would better capture the consequences of fiscal decentralisation for health outcomes. As this study used multisource data and not all data sources are available annually (for instance, Podes data only available 3 years), a multi-year study needs to consider other data sources. Moreover, the data we used to discuss child immunisation status was based purely on the verbal responses of parents who were not obliged to show an immunisation card, and whose answers regarding the completeness of their children's immunisation may have been influenced by recall bias. Future data collection is needed to improve measurement of individual past experiences.

Despite these limitations, our findings have several important implications. Firstly, this research indicates that districts continue to vary both in terms of immunisation coverage and also in terms of the extent to which local governments take advantage of the opportunities offered by fiscal decentralisation. While earlier studies focus on variation across countries, this study finds variation across districts and within one country, with the suggestion that the consequence of decentralisation on health status are more accurately assessed when districts and children are used as units of analysis. Secondly, this study extends the previous fiscal decentralisation measurements by referring only the resources allocated to healthcare services. One advantage of this measurement is that it better reflects responsible governance at the local level.

## Conclusions

Fiscal decentralisation is often promoted as a strategy to improve the performance of healthcare services, which in turn improve health outcomes, including immunisation status among children. However, the evidence across countries has not been definitive. This study has found that the transfer of fiscal authority to local governments is not a panacea of the problems of how to improve child immunisation status in Indonesia. Merely increasing the health budget at district level is not adequate. A new understanding is made possible here by investigating the regional disparities of public health programmes. The immense variation of immunisation coverage across districts suggests that lessons can be learned from the better-performing districts. Perhaps most significantly, in addition to increasing the discretion of local governments over decentralised funds, for fiscal decentralisation to be successful it demands a higher capability of local governments in order to deliver efficient and equitable public health services.

## Footnotes

1 The integrated health service post is a centre for children under five, women, and pre- and postnatal healthcare.

2 Children aged 12–23 months should receive one dose of BCG, three doses of polio, three doses of DPT, three doses of hepatitis B and one dose of measles vaccine (Table 1). The DPT, polio, and measles immunisation boosters are recommended after the child reaches 2 years. By their first birthday children should have completed the schedule, and another year is added to cover for possible delays.

## Appendix

**Appendix 1:** Determinants of child's immunisation status: proportion local health expenditure as tertile and quintile

	Model 4	Model 5
Household-level variables		
Residential areas		
(Rural)		
Urban	0.12 (0.04)‡	0.12 (0.04)‡
Birth attendants		
(Non-health professional)		
Health professional	0.40 (0.04)‡	0.40 (0.04)‡
Mothers’ employment status		
(Unemployed)		
Employed	−0.03 (0.03)	−0.02 (0.03)
Mothers’ age		
(≤20 years)		
21–30 years	0.13 (0.06)†	0.13 (0.06)†
>30 years	0.14 (0.07)†	0.13 (0.07)‡
Mothers’ education		
(Primary or less)		
Secondary	0.18 (0.04)‡	0.18 (0.04)‡
Higher	0.30 (0.06)‡	0.30 (0.06)‡
Log households income	0.18 (0.03)‡	0.18 (0.03)‡
District-level variables		
(Local health expenditure as a proportion of total expenditure (lowest tertile))		
Local health expenditure as a proportion of total expenditure (middle tertile)	0.16 (0.12)	
Local health expenditure as a proportion of total expenditure (highest tertile)	0.19 (0.12)	
(Local health expenditure as a proportion of total expenditure (lowest quintile))		
Local health expenditure as a proportion of total expenditure (second quintile)		−0.01 (0.16)
Local health expenditure as a proportion of total expenditure (middle quintile)		−0.00 (0.16)
Local health expenditure as a proportion of total expenditure (fourth quintile)		0.24 (0.15)
Local health expenditure as a proportion of total expenditure (highest quintile)		0.06 (0.16)
Proportion of urban population	0.54 (0.23)†	0.54 (0.23)†
Population density	−0.00 (0.00)‡	−0.00 (0.00)‡
Log GDP per capita	−0.14 (0.07)	−0.13 (0.07)
Between district variance	1.04	1.04
ICC	0.24	0.24
MOR	2.65	2.65

Note: Reported are marginal effects (standard error). Sig.:

* significant at 10% or less

† significant at 5% or less

‡ significant at 1% or less.

## Appendix

**Appendix 2:** Determinants of child's immunisation status: before and after multiple imputation

	Before multiple imputation			After multiple imputation		
		
	Model 1	Model 2	Model 3	Model 1	Model 2	Model 3
Intercept	0.05 (0.02)‡	0.02 (0.01)‡	0.01 (0.01)‡	0.04 (0.02)‡	0.02 (0.01)‡	0.01 (0.01)‡
Household-level variables						
Residential areas						
(Rural)						
Urban	1.12 (0.04)‡	1.13 (0.05)‡	1.13 (0.05)‡	1.13 (0.04)‡	1.12 (0.05)‡	1.12 (0.05)‡
Birth attendants						
(Non-health professional)						
Health professional	1.53 (0.06)‡	1.52 (0.06)‡	1.53 (0.06)‡	1.57 (0.06)‡	1.57 (0.06)‡	1.56 (0.06)‡
Mothers’ employment status						
(Unemployed)						
Employed	0.96 (0.03)	0.97 (0.03)	0.96 (0.03)	0.94 (0.03)	0.94 (0.03)	0.94 (0.03)*
Mothers’ age						
(≤20 years)						
21–30 years	1.13 (0.07)†	1.14 (0.07)†	1.13 (0.07)†	1.14 (0.07)†	1.14 (0.07)†	1.14 (0.07) †
>30 years	1.14 (0.07)†	1.14 (0.07)†	1.14 (0.07)†	1.14 (0.07)†	1.14 (0.07)†	1.14 (0.07)†
Mothers’ education						
(Primary or less)						
Secondary	1.19 (0.04)‡	1.19 (0.04)‡	1.20 (0.04)‡	1.19 (0.04)‡	1.19 (0.04)‡	1.19 (0.04)‡
Higher	1.36 (0.08)‡	1.35 (0.08)‡	1.36 (0.08)‡	1.35 (0.08)‡	1.35 (0.08)‡	1.35 (0.08)‡
Log households income	1.20 (0.04)‡	1.20 (0.04)‡	1.20 (0.04)‡	1.22 (0.04)‡	1.20 (0.04)‡	1.22 (0.04)‡
District-level variables						
Local health expenditure as a proportion of total expenditure (%)		7.68 (12.03)			8.56 (14.40)	
Hospitals/1,000 population			0.52 (1.16)			0.44 (1.02)
Health centres/1,000 population			0.65 (0.18)			0.64 (0.18)
Village health posts/1,000 population			1.71 (0.16)‡			1.88 (0.18)‡
Proportion of urban population		1.68 (0.39)†	2.10 (0.59)‡		1.91 (0.46)‡	2.47 (0.71)‡
Population density		0.99 (0.00)‡	0.99 (0.00)†		0.99 (0.00)‡	0.99 (0.00)†
Log GDP per capita		0.87 (0.06)*	0.88 (0.06)*		0.93 (0.71)	0.92 (0.07)*
Between district variance	1.09	1.05	1.01	1.20	1.20	1.10
ICC	0.25	0.24	0.23	0.27	0.27	0.25
MOR	2.71	2.65	2.61	2.85	2.84	2.72

Note: Reported are marginal effects (standard error). Sig.:

* significant at 10% or less

† significant at 5% or less

‡ significant at 1% or less.
